# Spectroscopic Techniques for the Characterization of Polymer Nanocomposites: A Review

**DOI:** 10.3390/polym10010007

**Published:** 2017-12-22

**Authors:** Liliane Bokobza

**Affiliations:** 196 Boulevard Bineau, 92200 Neuilly-Sur-Seine, France; Liliane.Bokobza@wanadoo.fr; Tel.: +33-1-4637-2427

**Keywords:** nanocomposites, spectroscopy, polymer-filler interface, fluorescence, NMR, infrared, Raman

## Abstract

Due to the growing interest in nanocomposites, a molecular characterization of these materials is essential for the understanding of their properties and for the development of new materials. Spectroscopic techniques that bring information at a molecular level are unavoidable when characterizing polymers, fillers and composites. Selected examples of the application of fluorescence, solid-state nuclear magnetic resonance (NMR), infrared and Raman spectroscopies, illustrate the potential of these techniques for the analysis of the filler surface, the evaluation of the state of filler dispersion in the host matrix, the extent of interaction between the polymer and the filler particles or the dynamics of polymer chains at the polymer–filler interface.

## 1. Introduction

Nanometer-scale particles such as spheres, plates and rods dispersed in a host polymeric medium have generated intense research in the development of rubber nanocomposites because they have been shown to significantly enhance the mechanical, electrical and thermal properties of the pristine polymer. These nanoparticles are expected to yield a high interfacial area between the organic and inorganic phases only in the case of a good dispersion of the filler particles within the polymeric matrix. Another important factor that assists filler dispersion and determines the level of matrix reinforcement, is the interfacial bonding between the two phases [1,2].

Static and dynamic mechanical behaviors can reveal the importance of interfacial adhesion and filler dispersion. In conventional composites, such as those filled with silica or carbon black, high elastic modulus and high tensile strength are obtained in the case of a strong interface, while a strong increase in the composite initial modulus followed by a slight increase in stress in a large range of deformations are observed in the case of a weak interface. For example, a weak interface is displayed by silica-filled hydrocarbon rubbers, but the introduction of a chemical coupling agent in the filled system increases the adhesion between the filler and the rubber and consequently the reinforcing capability of silica [3]. The steep increase in the elastic modulus observed at low deformations in the absence of polymer–filler interactions, is attributed to agglomerated filler structures yielding at high filler contents the formation of a filler network throughout the polymer matrix. This effect, which may be regarded as a result of poor dispersion, is better visualized through the dynamic mechanical properties of the composites, where it can be seen that the strain dependence of the storage modulus exhibits a strong non-linear behavior known as the Payne effect. The Payne effect has been attributed to the breakdown of filler agglomerated structures, since it has been observed at filler contents below the percolation threshold [2].

Nowadays, nanosized inorganic structures can be generated in situ in rubbers, thermoplastics and thermosets via a sol–gel route consisting of hydrolysis and the condensation of inorganic alkoxides of metals like Si, Ti, Al, Zr, Ta, Sn, Hf, … [4,5,6,7,8,9,10] in the presence of a catalyst. This process allows the precipitation of a mineral phase more finely dispersed than in the case of a mechanical mixture. On the other hand, this process allows a tailored manipulation of the organic and inorganic phases at the nanometer length scale by a control of the processing conditions. The resulting composites exhibit excellent mechanical and thermal responses as well as optical transparency. The high level of dispersion combined with the small size of the particles, offers a large interfacial area available for polymer–filler interactions. In the case of poly(dimethylsiloxane) (PDMS) networks filled with in-situ-generated silica particles, these interactions are ensured via hydrogen bonds between the hydroxyl groups present on the silica surface and the oxygen atoms of the polymer chains [11,12]. Silica-titania mixed-oxide fillers precipitated into PDMS networks have been shown to impart to the elastomeric matrix higher modulus and higher extensibility than networks filled with a single filler [13,14].

The production of nylon 6/clay nanocomposites by the Toyota Research Group, has initiated intense research activities into composites based on layered silicates consisting of stacked units of one to a few nanometers thick. The main idea is to obtain an exfoliated or delaminated structure in which the silicate layers are homogeneously dispersed in the host matrix, which can only be obtained if the layered clays are rendered compatible with the organic polymer by ion-exchange reactions with cationic surfactants such as alkylammoniums [15,16,17,18,19]. Clay exfoliation in a polymer matrix is expected, by separating the different layers, to give rise to an enormous interface area, leading to outstanding improvements in the mechanical, thermal and barrier properties. One major advantage of exfoliated nanocomposites is to decrease the permeability to gas and water vapor, making them particularly useful in food packaging [20]. These gas barrier properties, ascribed to the high aspect ratio of the nanolayers, are connected to the degree of exfoliation and dispersion of the clay platelets, which depends on the processing conditions. Clay nanocomposites are usually formed through an in situ polymerization process or by using conventional melt processing techniques such as extrusion or injection molding. One drawback of clay nanocomposites is their low electrical conductivity, which can be overcome by adding to clay a small amount of carbon-based materials well known to be conductive inclusions. The dual filling approach that relies on possible synergistic effects between two different filler morphologies, namely spheres and rods [21] or spheres and plates [22], has proven to yield a better physical performance of the resulting composite.

Carbon nanomaterials, including carbon nanotubes, graphite or graphene, are now well known to provide to insulating polymers an electrical conductivity at lower filler contents than conventional carbon blacks [2]. Besides their outstanding electrical properties, their high aspect ratios and exceptional mechanical properties made them considered as advanced reinforcing fillers for polymeric matrices. Among these carbon nanostructures, carbon nanotubes (CNTs) have attracted enormous interest due to their unique structure consisting of cylinders of one or more graphene sheets. For example, at a same filler loading, multiwall carbon nanotubes (MWCNTs) dispersed in a styrene–butadiene rubber (SBR), have been shown to impart to the elastomeric matrix much higher stiffness than carbon black or silanized silica, but the deformation at break decreases with the filler loading as the result of the presence of agglomerates acting as failure points [2]. A strong Payne effect increasing with the nanotube content also reflects a poor dispersion of MWCNTs in the host matrix. Almost all studies report the poor dispersion of CNTs in polymeric media due to a lack of interfacial adhesion and to the tendency of the tubes to bundle together. The functionalization of the tube surface is intended to bring some adhesion between the tube and the polymer, thus enabling effective stress transfer at the polymer–filler interface. Appropriate functionalization of the tube surface or introduction of a coupling agent able to react with both phases has been shown to achieve good dispersion and good interaction with the polymer chains [23]. A recent review by Punetha et al. [24] compiles recent developments in the aspects of the functionalization of carbon nanomaterials for advanced polymer nanocomposites. It includes discussion of the methods and various techniques used for the functionalization of carbon nanomaterials, both covalent and non-covalent, along with their crucial applications. Zhang et al. [25] have shown that, besides improving the stability and ability to disperse and thus processibility, the functionalization of carbon-based nanomaterials with photochromic molecules, including azobenzenes, diarylethenes, spiropyrans/-oxazines and stilbenes, imparts them with enhanced/additional properties, making them novel multifunctional responsive materials. Fullerenes, carbon nanotubes and graphene functionalized with photochromic molecules can exhibit reversible changes triggered by light.

As for layered silicates, the layered structure of graphite has the ability to be intercalated and exfoliated. Total exfoliation into individual layers is a major challenge and various preparation techniques aimed at obtaining single sheets have been developed through graphite intercalation compounds, expanded graphite or graphite oxide by using chemical, mechanical or electrochemical techniques. These techniques often lead to stacks of graphene that are 1 to 10 nm thick, called graphite nanostructures or graphite nanoplatelets (GNPs). The chemical conversion of graphite into graphene oxide, intended to yield the large-scale production of graphene, in fact generates a lot of structural defects, as revealed by Raman spectroscopy [26,27,28,29]. The resulting oxidized material cannot be used as a conductive filler, because it displays low electrical conductivity due to a disruption of its conjugated electronic structure. The reader will find further details on the production of the different graphite nanostructures and on the processing conditions and characterization of graphite- and graphene- based nanocomposites in the literature [30,31,32,33,34,35,36,37,38,39]. Strong reinforcing effects have been obtained by filling polymers with graphitic nanomaterials [2]. The improvements, highly dependent on the state of filler dispersion, have been essentially attributed to the particle morphology rather than to strong interfacial effects.

All of the above considerations show that the key parameter of filled system performance is the polymer–filler interface that largely governs the mechanical properties of the composite material. The results often do not completely meet the expectations, because the lack of interfacial adhesion yields a poor dispersion system in which the presence of agglomerated filler structures negatively affects the rupture properties of materials. Besides the macroscopic information provided by a mechanical analysis, a molecular level characterization provided by molecular spectroscopy is required for an identification of the interacting species, which allows a better understanding of the composite properties and helps the development of new polymer composites by making more efficient the processing conditions.

This review is intended to illustrate some applications of molecular spectroscopy (fluorescence, solid-state NMR, infrared and Raman spectroscopy) for the analysis of polymer composites and also to give examples of the combination of atomic force microscopy and infrared spectroscopy (AFM-IR) and tip-enhanced Raman scattering (TERS) to get chemical information with nanometric spatial resolution bringing new insights into the sample structure by surpassing the diffraction limit of traditional vibrational microspectroscopy. The fundamental principles of the various molecular spectroscopies and the information provided by these characterization techniques, can be found in reference [40] and in the references therein.

## 2. Fluorescence Spectroscopy

Investigations by fluorescence spectroscopy require the incorporation in the medium of a fluorescent probe used at a very low concentrations in such a way that the bulk is not excessively perturbed by the presence of the chromophores. The probe is chosen for its ability to detect changes in its immediate environment through the change in its emission behavior.

The analysis of the luminescence properties of a small probe incorporated into a polymer matrix has been shown to lead to valuable information on various phenomena of polymer science, including the dynamics of polymer chains through excimer fluorescence [41], phase separation and polymer miscibility, transport processes or polymer degradation [42]. The application of fluorescence studies to the analysis of polymer composites mostly exploits a particular photophysical phenomenon, like energy transfer or the quenching of fluorescence.

**Figure 1:**
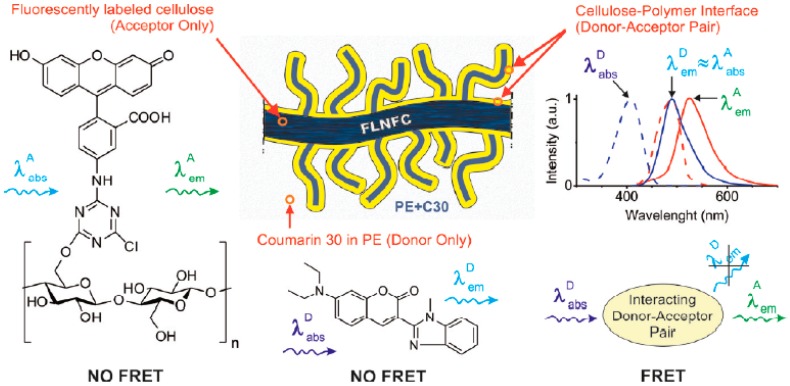
Schematic drawing illustrating the use of FRET for revealing the interface in polymer composites. Reprinted from reference [43] with permission from ACS Publications.

Förster resonance energy transfer (FRET) has been used by Zammarano et al. [43] to monitor interface and dispersion in polymer nanocomposites. FRET is a mechanism that describes energy transfer between a donor fluorophore, in an electronic excited state, to an acceptor through nonradiative dipole–dipole coupling. FRET requires an overlap between the fluorescence spectrum of the donor and the absorption spectrum of the acceptor. Moreover, the efficiency of the FRET process depends on the distance between the two molecules, which should be in close proximity, typically at a distance from 1 to 10 nm. In the field of polymer nanocomposites, FRET can be applied to reveal nanofeatures occurring at the interface of a polymer–filler system provided that both phases are labelled with suitable donor–acceptor chromophores. In the work of Zammarano et al. [43], nanofibrillated cellulose (NFC) fluorescently labelled with 5-(4,6-dichlorotriazinyl) aminofluorescein (FL) (the acceptor) is dispersed into polyethylene doped with Coumarin 30 (C30) (the donor) and FRET is used to evaluate the dispersion of the reinforcing phase. A higher extent of dispersion generates an increase in interface and thus in FRET. Figure 1 illustrates the use of FRET for revealing the interface in polymer composites and shows that interacting donor–acceptor pairs should be formed to allow energy transfer.

The fluorescence behavior of optical probes (Nile Blue A and methylene blue) in polystyrene and polyamide-6, has been shown to be a potential tool for the analysis of intercalation and exfoliation in melt-processed polymer clay nanocomposites [44].

**Figure 2:**
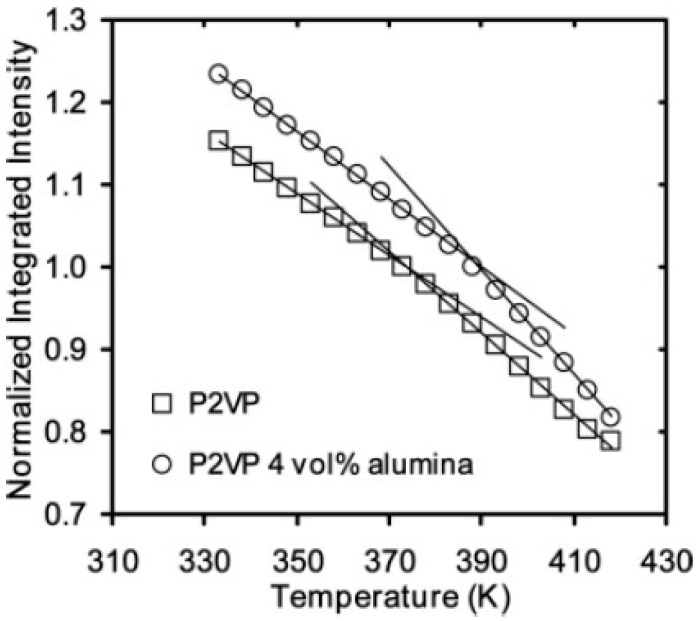
Temperature dependence of the normalized integrated intensity of the pyrene dopant (<0.2 wt %) in a bulk poly(2-vinyl pyridine) P2VP film and in a 4 vol·% alumina/P2VP nanocomposite film. (The integrated intensity has been normalized to one at *T*_g_). Reprinted from reference [45] with permission from Wiley.

The fluorescence of dyes (pyrene, 1,10-bis(1-pyrene) decane (BPD) or 4-tricyanovinyljulolidene) (TCJ) was used to study the effects of confinement on the glass transition temperature (*T*_g_) and physical aging in polystyrene (PS), poly(methyl methacrylate) (PMMA) and poly(2-vinyl pyridine) (P2VP) nanocomposites containing 10- to 15-nm-diameter silica nanospheres or 47-nm-diameter alumina nanospheres [45]. The dye, incorporated in the filled system, exhibits a different temperature-dependence of its fluorescence intensity below and above *T*_g_ and the intersection of the two dependencies is *T*_g_, as shown in Figure 2. A substantial, 16 K increase in *T*_g_ of the P2VP/4 vol·% alumina nanocomposite is observed, relative to neat, bulk P2VP. It is attributed by the authors to strong attractive interactions between the two phases, leading to reductions in cooperative segmental mobility in the polymer and an increase in *T*_g_. In contrast, no change in *T*_g_ has been observed in PS–alumina nanocomposites, which has been presumably ascribed to polymer–filler interfaces lacking attractive interactions. More interestingly, the use of the TCJ dye that acts as a “rotor probe” for monitoring the physical aging rate, leads the authors to conclude that physical aging is strongly reduced in the composite.

An interesting application of fluorescence spectroscopy concerns the investigation of the stress-softening phenomenon in filled elastomers, known as the Mullins effect and characterized by a pronounced lowering in stress when the material is stretched a second time after the release of the first load. Over the past decades, the Mullins effect has generated great interest, with an attempt to understand the molecular origin of this effect. However, until now, there is no general agreement on the origin of this effect, most probably because different mechanisms depending on the characteristics of the polymer–filler system, can be involved in this particular phenomenon. In the work of Clough et al. [46], mechanically-induced chemiluminescence or mechanoluminescence has been used to demonstrate that covalent bond scission contributes significantly to the stress-softening effect. Bis(adamantyl)-1,2 dioxetane (mechanophore) contained within the cross-linker and 9,10-diphenylanthracene (DPA) were incorporated in silica-filled poly(dimethylsiloxane) (PDMS). The mechanophore cleaves upon the application of a force, to give excited ketones, which relax to the ground state with the emission of light. This emission, of low intensity, is transferred to DPA, which acts as a fluorescent acceptor molecule via a FRET mechanism.

## 3. Solid-State NMR Spectroscopy

One of the major advantages of solid-state NMR is to allow the analysis of polymer–filler interfaces due to the sensitivity of the NMR spectra and the relaxation parameters to the local and segmental molecular motions of polymer chains. Polymer–filler interactions usually contribute to the formation of an adsorption layer where chain motions are more restricted than those in the bulk, and solid-state NMR has been shown to be able to differentiate the polymer behavior in the interfacial region from that in the bulk.

**Figure 3:**
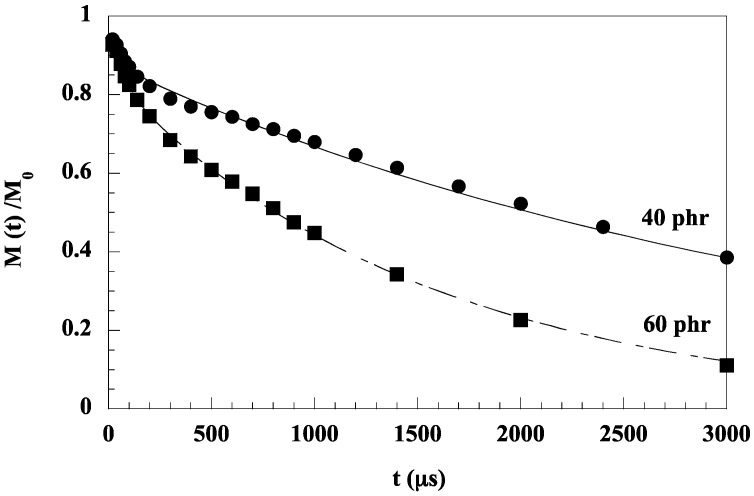
Proton NMR relaxation data for tin-catalyzed silica-filled poly(dimethylsiloxane) (PDMS/SiO_2_) composites. Reprinted from reference [11] with permission from Elsevier.

In PDMS networks filled with in-situ-generated silica particles, two different spin–spin relaxation times, *T*_2_, related to the polymer in the bulk (*T*_2_^mob^) and to the polymer at the interface (*T*_2_^rig^), were extracted from the transverse magnetization relaxation function (Formula (1)) in solid state proton NMR studies using spin–echo techniques [11] (Figure 3):

*M*(t) = *M*_0_ exp [−*t*/*T*_2_^mob^] + (1 − *M*_0_) exp [−*t*/*T*_2_^rig^] (1)

In Formula (1), *M*_0_ represents the fraction of mobile chains outside the adsorption layer whose dynamics are expected to be close to those of the unfilled rubber, and *T*_2_^mob^ and *T*_2_^rig^ are the spin–spin relaxation times of the corresponding components. These measurements can allow an estimation of the thickness of the interfacial layer, i.e., by the following expression:

(2) $$e=R\left[{\left(1+\frac{\omega (1-\phi )}{\phi }\right)}^{1/3}-1\right]$$

where ω is the fraction of immobilized polymer and φ, the volume fraction of filler and R the radius of the particles. In the work of Dewimille et al. [11], the thickness of the adsorption layer has been estimated around 1.5 nm.

*T*_2_ measurements under external mechanical stress and at high temperatures in the melt (up to 250 °C) were carried out by Böhme and Scheler [47] in a poly(propylene) and a poly(propylene nanocomposite). The relaxation time exhibits the expected two-component decay, where the short decay fraction is associated with the part of the polymer experiencing restricted motion. In the pure polymer, the longer component becomes significantly longer at elevated temperatures indicating increased polymer chain mobility. In the nanocomposite, the effect of the increase in temperature is less pronounced and the longer decaying fraction shows a shorter *T*_2_ than in the pure polymer. The authors conclude that the interaction with the filler leads to an overall restriction of the motion that extends beyond the directly interacting polymer chains.

**Figure 4:**
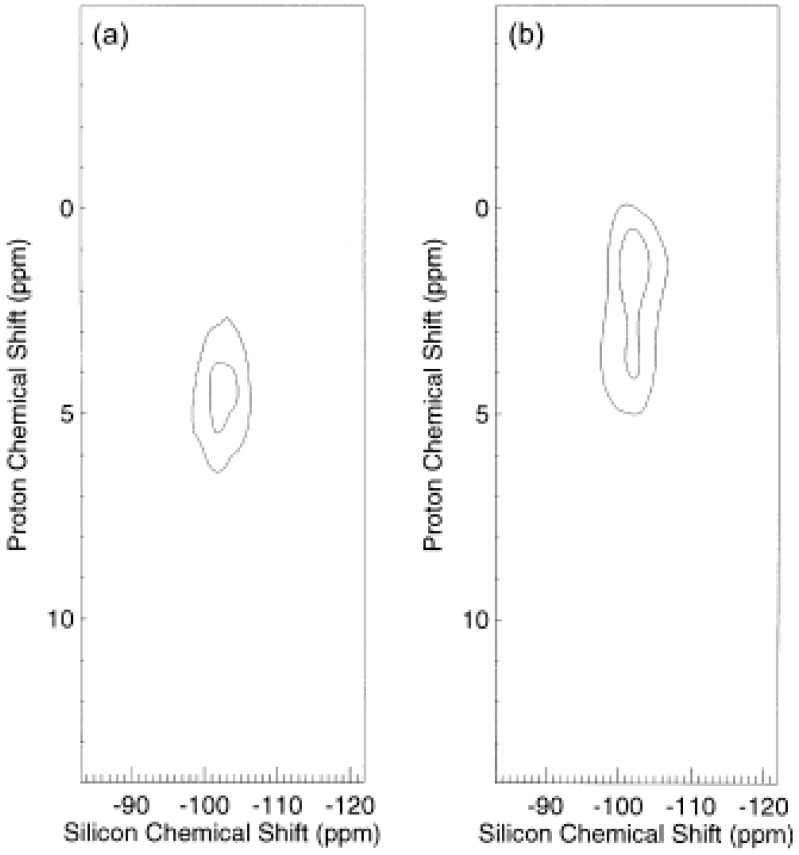
The two-dimensional 2D proton-silicon heteronuclear correlation spectra of the poly(ethylacrylate)/vycor composite obtained with a spin diffusion delay time of (**a**) 50 μs and (**b**) 50 ms. Reprinted from reference [49] with permission from Elsevier.

In a chapter devoted to the solid-state NMR characterization of polymer interfaces, Mirau [48] describes, besides solid-state proton NMR, other NMR methods to study the molecular dynamics of polymers. Proton–proton double quantum correlation spectroscopy, heteronuclear correlation NMR or solid-state deuterium quadrupolar-echo spectra, have been shown to probe the polymer properties at the interface. Proton–silicon heteronuclear correlation experiments with two different spin diffusion times have been used to characterize the interface of composites based on poly(ethylacrylate) filled with vycor, which is a porous glass (Figure 4). Figure 4a displays a correlation between protons at 4.5 ppm (assigned to hydroxyl protons and a layer of water molecules at the surface of the pores of vycor) and the *Q*_3_ sites of the vycor silicon surface. A correlation between the *Q*_3_ silicon and proton peaks at 1.3 and 4 ppm of the polymer (Figure 4b) is established with a longer spin diffusion delay [49]. The authors conclude that the polymer occupies the center of the pore and is insulated from the silica surface by a layer of water. It has to be mentioned that solid-state ^29^Si MAS NMR has been shown to be well-suited to a detailed analysis of the surface silanol hydroxy groups (isolated and geminal, also denoted *Q*_3_ and *Q*_2_, *Q*_4_ being related to SiO_4_ species) located between 90 and 120 ppm [50].

Solid-state NMR was also performed on poly(methylmethacrylate) (PMMA)-based hybrids containing neat and modified (mSiO_2_) silica nanoparticles [51]. Methacryloylpropyltrimethoxysilane (MPTMS) was selected as the surface modifier and its vinyl end groups can participate in acrylic radical polymerization, giving rise to an interphase consisting of PMMA chains grafted onto the silica surface, mSiO_2_-*g*-PMMA. ^29^Si solid-state NMR has been used to characterize SiO_2_ and mSiO_2_ and measurements of the relaxation times in the rotating frame showed slightly longer values in the mSiO_2_-*g*-PMMA phase than in the neat polymer, indicating a small variation in the dynamic induced by the grafting of PMMA on modified silica particles. However, the *T*_g_ value of PMMA in nanocomposites containing neat and modified silica show an increase of 6 and 12 °C, respectively, with respect to PMMA.

Polymer/clay nanocomposites have also been characterized by solid-state NMR spectroscopy in order to study the phase structure and chain dynamics in the exfoliated and intercalated nanocomposites, changes in the molecular mobility, or the interactions between the two components [52,53,54]. ^13^C and ^1^H were used to analyze the segmental dynamics of poly(ethylene oxide) (PEO) chains in a model polymer/laponite intercalated phase [55]. Laponite, which is a phyllosilicate consisting of platelets with a discoic shape, was used at a high content (70 wt %) in order to obtain a neat intercalated phase in which the PEO chains are all amorphous. A significant slowing down of the segmental motions of the amorphous phase was observed and was mainly assigned to the complexation of PEO oxygens by the Na^+^ counterions located in the laponite interlayer galleries.

**Figure 5:**
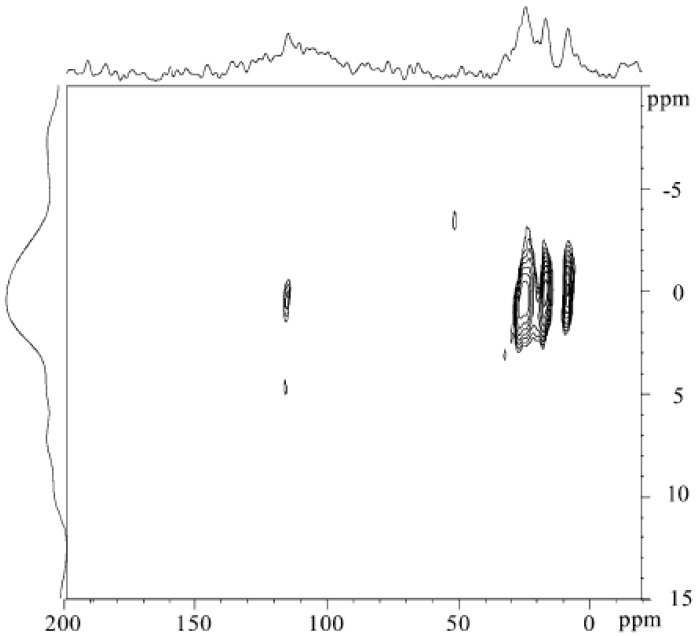
^1^H–^13^C 2D correlation spectra of functionalized carbon nanotubes. Reprinted from reference [56] with permission from the American Chemical Society.

In the work of Cahill et al. [56], dedicated to the analysis of functionalized carbon nanotubes with polymers such as PMMA, fast magic-angle spinning (30 kHz), applied to achieve high-resolution ^1^H-NMR, together with advanced pulse sequences such as ^1^H double quantum NMR with the BABA (back-to-back) sequence, and heteronuclear ^1^H–^13^C sequences, were used to confirm the functionalization process and establish the correlation of the aliphatic protons with the carbon nanotubes. Figure 5 represents the ^1^H–^13^C 2D correlation spectra showing a clear correlation between the ^13^C resonance of the nanotube (at 121 ppm) and the ^1^H resonance of the aliphatic functional group (at 0.5 ppm), thus providing evidence of the interaction between the grafted group and the nanotube surface.

## 4. Infrared and Raman Spectroscopy

Infrared absorption and Raman scattering are molecular spectroscopies widely used to obtain information on polymeric systems from their vibrational properties. While infrared radiation arises from a direct resonance interaction between the frequency of the infrared incident radiation and that of a particular vibrational mode, the Raman effect is an inelastic scattering of light occurring upon the irradiation of a molecule with a monochromatic light (usually a laser). One major advantage of Raman scattering is to allow the analysis of thick polymer samples while only very thin films can be examined by infrared transmission spectroscopy, since infrared radiation is readily absorbed by functional groups of the polymer.

The vibrational spectra of polymer composites can be used to identify bands associated with vibrational modes of functional groups of both polymer chains and filler particles. The analysis of vibrational spectra can provide information on the interaction between the organic and inorganic phases, the state of intercalation and exfoliation in polymer nanocomposites containing layered silicates, filler dispersion and functionalization, the degree of orientation of both the polymer chains and the reinforcing anisometric particles.

### 4.1. Infrared Spectroscopy

**Figure 6:**
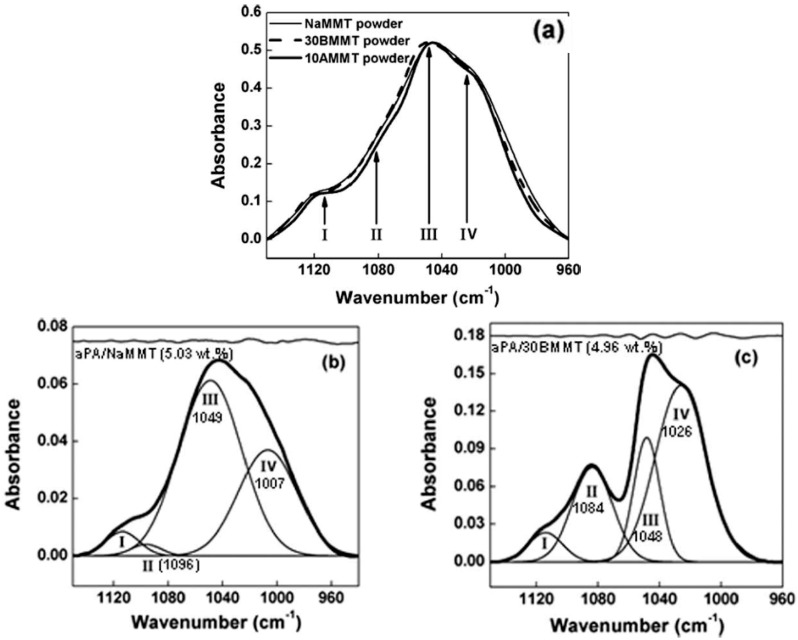
Fourier transform infrared FTIR spectra of: (**a**) sodium montmorillonite (NaMMT) and organoclays (30BMMT) and (10AMMT) respectively intercalated with methyl, tallow, bis-2-hydroxyethyl quaternary ammonium and dimethyl, benzyl, hydrogenated tallow quaternary ammonium; (**b**,**c**): clays in aPA composites. Reprinted from reference [58] with permission from Wiley.

The potential of infrared spectroscopy as a method for characterizing the state of intercalation/exfoliation in polymer nanocomposites containing montmorillonites has been clearly demonstrated by Cole [57] by using the change in the clay Si–O band envelope. It was shown that the shape of the clay absorption envelope between 1350 and 750 cm^−1^ changes as a function of processing, resulting presumably from better intercalation and exfoliation. However, as the polymer composites were processed with a compatibilizing agent, according to the author, interactions between this agent and the Si–O dipoles of the nanoclay can also be partly responsible for the observed results. Subsequently, Zhang et al. [58] also use Fourier transform infrared (FTIR) spectroscopy to characterize the state of dispersion of layered silicates in polymer nanocomposites based on poly(hexamethylene isophthalamide) (aPa) and montmorillonite nanoclay (MMT). The Si–O band envelope of sodium montmorillonite (NaMMT) and two organoclays, displays four usual components (Figure 6a). The spectral pattern of the unmodified clay particles (strongly agglomerated as revealed in the TEM image) in the polymer (Figure 6b) is broader and has a lower intensity than that of the delaminated organoclay in the same matrix (Figure 6c). Peak II (associated with the out-of-plane vibration mode of the Si–O bond) shows a substantial increase in intensity and shifts towards lower wavenumbers, while peak IV (associated with the in-plane vibrational mode) shifts to higher wavenumbers in the exfoliated structure. It appears that the clay Si–O band envelope can be used as an indicator of clay dispersion in a host polymeric matrix.

**Figure 7:**
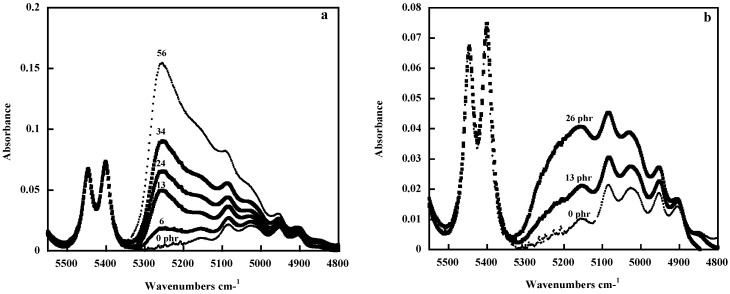
Near-infrared spectra in the 4800–5600 cm^−1^ range of pure PDMS and of PDMS/SiO_2_ (**a**) and PDMS/TiO_2_ (**b**) composites: each curve is labelled with the amount of filler in phr (parts per hundred parts of rubber). Reprinted from reference [59] with permission from Wiley.

Infrared spectroscopy has been used to characterize water at the silica (or titania)-polymer interface [59]. Water molecules insulate the polymer from the filler surface, which could be detrimental for the formation of a strong interface. The hydroxyl groups present on the particle surface can be linked to water molecules, which can be characterized by their O–H stretching band around 3700–3000 cm^−1^ and the H–O–H bending band at 1640–1630 cm^−1^. These bands are usually strong in thick samples analyzed in transmission in the classical mid-IR region. However, the combination of the bending and the stretching modes, which are located in the 5300–4800 cm^−1^ range and much weaker than the corresponding fundamental absorptions, can be easily identified in the near-infrared (NIR) range of the spectrum. As seen in Figure 7, which represents the NIR spectra of PDMS filled with silica (a) and titania (b) particles, the absorptions associated with the water molecules increase in both cases with the amount of filler, but the two types of composites display a different spectral pattern revealing a different water interface. This can be explained by the fact that the two types of particles exhibit two distinct morphologies [12]; the more condensed titania particles make possible a clustering of water around the first adsorption site, rather than the open-fractal structure of the silica particles.

The surface modification of carbon nanotubes during the functionalization process intended to improve the compatibility of the carbon material with the polymer matrix, is usually followed by infrared spectroscopy [60,61,62]. In the work of Lee et al. [63], the CNT surface was modified by three methods: acid, heat and hydrogen peroxide treatments. The strong intensity of the band located at 1089 cm^−1^ in the infrared spectrum of H_2_O_2_ treated CNTs, reflects the presence of many C–O bonds generated during hydrogen peroxide treatment. This treatment has been shown to improve the thermal stability of poly(amide-imide)/CNT composites.

Infrared spectroscopy studies of poly(vinylpyrolidone) (PVP) containing gold nanoparticules reveal interactions between the C=O groups of PVP and metal particles [64]. However, a FITR investigation of polyrethane containing a dual filling of gold nanoparticles and graphene intended to bring electrical conductivity to the matrix, did not provide evidence of strong interactions between the polymer chains and the incorporated particles [65]. The addition of Ag nanoparticles into a polyaniline/diamond/functionalized MWCNTs was characterized by the occurrence of a band at 1036 cm^−1^ indicating an interaction between polyaniline and silver nanoparticles [66].

The orientation of polymer chains taking place on deforming uniaxially-unfilled and -filled elastomeric networks can be detected and quantified by infrared spectroscopy. The analysis of the orientation behavior of filled networks, which is only applicable to systems filled with a non-black filler, has allowed an evaluation of the degree of bonding between the polymer and the filler [3,67,68]. The segmental orientation in a uniaxially oriented sample may be conveniently described by the second Legendre polynomial:

(3) $$<P_{2}(\cos\theta )>=\frac{2}{3\cos^{2}\beta -1}.\frac{R-1}{R+2}$$

where θ is the angle between the direction of extension and the local chain axis of the polymer, β is the angle between the transition moment vector of the vibrational mode considered and the local chain axis of the polymer or any directional vector characteristic of a given chain segment and R (R = *A***_//_**/*A***_⊥_**) is the dichroic ratio (*A***_//_** and *A***_⊥_** being the absorbances of the investigated band, measured with radiation polarized parallel and perpendicular to the stretching direction, respectively).

As a typical example, in the case of a PDMS film of about 2 mm in thickness, due to the strong intensity of most of the bands associated with the fundamental modes, the much weaker overtones and combination bands can be used to evaluate chain orientation [69]. It was shown that the second moment of the orientation function increases with the filler content. <P_2_(cosθ)> was derived from the dichroic behavior of the band located at 2500 cm^−1^ ascribed to the overtone of the symmetrical bending vibration of the methyl group and whose transition moment is expected to make an angle of 90° with the vector joining two successive oxygen atoms chosen as a chain segment [69]. As the second moment of the orientation function has been shown to depend on the number of bonds between chemical junctions [70], the increase in orientation in filled materials has been ascribed to polymer–filler interactions, creating additional cross-links that increase with the amount of filler or with the interfacial area of the polymer–filler system. It becomes possible to determine the number of reactive sites per nm^2^ of filler surface, thus making infrared spectroscopy a suitable technique for quantifying the degree of bonding of the network chains to the filler surface.

Cole et al. [71] also use infrared spectroscopy and the “tilted film method” to determine the orientation of both polymer chains and clay platelets in blown films of composites based on polypropylene and clay particles. The clay orientation was found rather high, which was expected due to the high anisometric character of this type of filler in contrast to polymer chains that only exhibit a moderate level of orientation.

In an analysis of a polystyrene/carbon nanotube composite, no significant change in the degree of polystyrene molecular orientation was also observed by the addition of carbon nanotubes [72]. This is in contrast with the results reported above for the silica-filled PDMS networks where the chain orientation increases with the silica content due to a strong polymer–filler interface resulting from the interaction between the silanols present on the silica surface and the PDMS chains.

It is interesting to mention the AFM-IR technique that combines atomic force microscopy and infrared spectroscopy in order to obtain infrared spectra with a nanoscale spatial resolution as described in a recent review [73]. This technique overcomes the fundamental limit of conventional infrared spectroscopy, specifically the spatial resolution limits imposed by diffraction. AFM-IR has found applications in polymers, life sciences, photonics, solar cells, semiconductors or pharmaceuticals. Examples of the use of the AFM-IR technique in the field of polymer composites have been described in the work of Marcott et al. [74]. They include an isotactic poly(propylene) film with added SiO_2_ particles, a polymer with carbon black particles and a carbon-fiber/epoxy composite material. The AFM-IR technique can bring information on the interphase region between a particular nanomaterial inclusion and the bulk polymer. As already mentioned, characterizing the polymer–filler interface is crucial for the understanding the mechanical properties of the overall composite. In the example of carbon fiber/epoxy composite, spectra are recorded at different locations on the AFM image. Some spectral features collected close to the interface differ from those collected in the bulk, suggesting a different chemical nature of the polymer phase in the vicinity of the carbon fiber, which is consistent with nanoscale mechanical properties.

### 4.2. Raman Spectroscopy

The interest in Raman spectroscopy for the analysis of composites based on carbon materials has been revived in the last two decades with the advent of carbon nanotubes [75]. This non-destructive technique has been extensively applied for the characterization of the vibrational states of various carbon-based materials including diamond, graphite, graphene, fullerene and carbon nanotubes. As they display resonance-enhanced Raman scattering effects, they give rise to strong, well-defined bands even if they are dispersed in a polymer matrix at a very low content. This makes Raman spectroscopy one of the most important techniques for the analysis of composites containing carbon materials.

**Figure 8:**
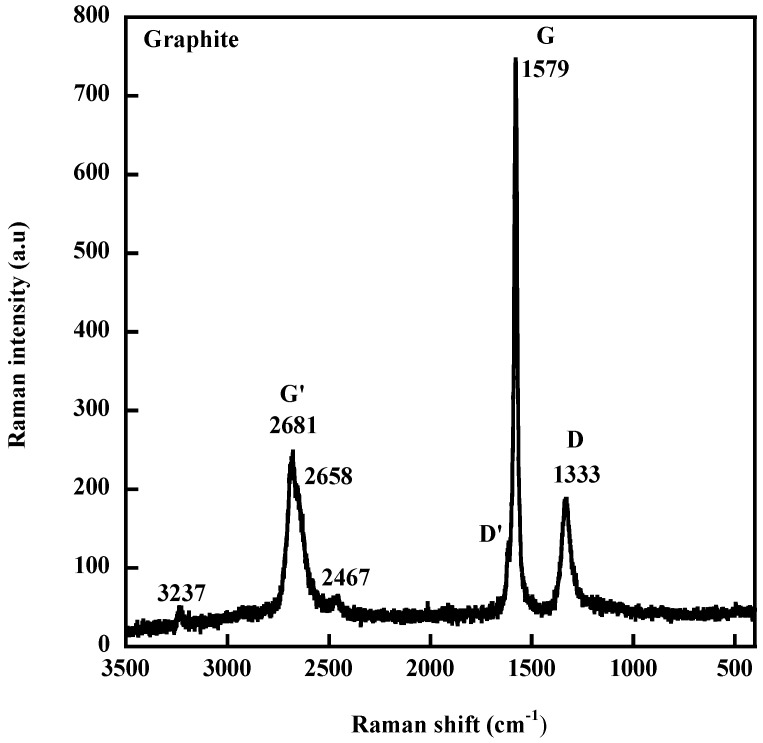
Raman spectrum of a graphite (Graphite 3775 from Asbury Carbons, a surface-enhanced flake graphite with a specific area of 24 m^2^·g^−1^) excited at 633 nm.

The basic principles of Raman spectroscopy as well as the specific features of the Raman spectra of carbons have been recalled in a recent review [76]. Very briefly, carbon materials examined at 633 nm, display, around 1580 cm^−1^, the G band typical for graphite crystal. In addition to the G band, which is a common feature of all carbon graphitic materials, Raman spectra display a second band (D band at 1333 cm^−1^ for a graphite represented in Figure 8 as a typical example) and a high frequency shoulder around 1600 cm^−1^ (D′ band). Both D and D′ bands are assigned to defects within the carbon structure (edges, distorded graphene layers). The disorder can also be quantified by measuring the I_D_/I_G_ ratio between the disorder-induced D band and the G band. One important feature in the Raman spectrum of carbon materials is the occurrence of a band around 2681 cm^−1^ (G′ band) corresponding to the first overtone of the disorder-induced D band and observable even in the absence of defects.

Several factors have been shown to affect the spectral features of carbon materials. They include temperature, pressure, strain, laser excitation energy, filler–filler and polymer–filler interactions, orientation and even functionalization [76,77]. However, as recommended by Everall et al. [78], for a correct interpretation of the Raman data, one has to take into account possible laser-induced sample heating that has been shown to significantly shift vibrational Raman modes of carbonaceous materials. The heating effects induced by laser irradiation of the sample yield an increase in the local temperature resulting in a shift to lower wavenumbers of the Raman bands both for pristine carbon materials and for the carbon species embedded in a polymeric matrix. The shift increases with the laser power, but as observed by several authors, the shift is lower for the carbon material in the composite than in the non-embedded state [77,78,79,80]. Kao and Young [79] attribute the lower sensitivity of the G′ band of SWNCTs incorporated at 0.1 wt % in an epoxy resin to the dispersion of the nanotubes in the matrix which makes them less prone to heating effects, or to the epoxy matrix, which limits the thermal expansion of nanotubes, causing an elongation of the C–C bonds. This factor is recognized as being mainly responsible for the shift of the Raman wavenumbers with the laser beam power. For styrene-butadiene (SBR) (a softer matrix than epoxy resin) filled with different amounts of MWCNTs (3, 5 and 10 phr), Yan et al. [80] also observed for all the samples, a shift of the G band to a lower wavenumber with an increase in the laser power. However, at the same laser power, the shift increased with the amount of filler and became close to that of pure MWCNTs. For the sample filled with 3 phr of MWCNTs, for example, the G band shifted by 9 cm^−1^, while that of pure MWCNTs was around 16 cm^−1^ at the laser power of 7 mW. The different shift behaviors of MWCNTs and of SBR/MWCNT composites were explained by a rearrangement of the carbon nanotubes in the composite or a mechanical compression induced by the SBR matrix, thus blocking the expansion of MWCNTs with increasing temperatures. In a Raman investigation of isotactic polypropylene/MWCNT composites (iPP/MWCNTs) at 0.25, 0.5, 1 and 4 wt %, Bounos et al. [77] observed for all the samples a linear downshift of the G band frequency with increasing laser power and for the same laser power, the shift increased with the MWCNT content in the polymer matrix. Moreover, at the same filler loading, but at two different states of dispersion, it was demonstrated that the shift of the G band was much more important for the poorly dispersed sample at a high laser power. For this reason, the authors conclude that the G-band frequency can be used for the characterization of the MWCNT dispersion and its shift is attributed to the local temperature of the measurement region. The main parameter responsible for the change in band frequency is, according to the authors, the temperature that increases due to the sample’s absorption, linked to the MWCNT content. It is considered that the temperature increase is a simple exponential function of the molar concentration of the absorbing species if the thermal conductivity of the composite is constant. The thermal conductivity of SBR/MWCNT composites has effectivity been shown to increase very slightly with the nanotube loading (0.12 W/mK for pure SBR and 0.20 for the sample filled with 10 phr of MWCNTs) [81] despite the very high thermal conductivity of individual nanotubes.

**Figure 9:**
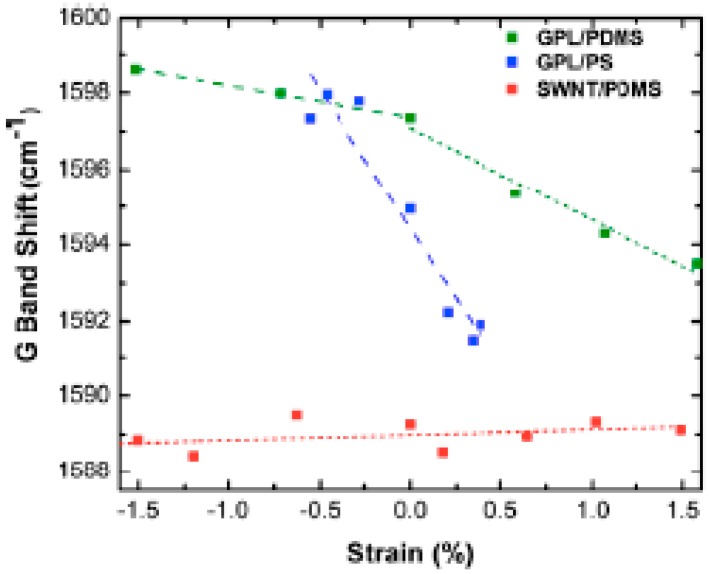
Comparison of Raman G-band peak shift in tension and compression of graphene platelets/poly(dimethylsiloxane) (GPL/PDMS), single-walled carbon nanotubes/poly(dimethylsiloxane) (SWCNT/PDMS) and graphene platelets/ polystyrene (GPL/PS) nanocomposites below 2% applied strain at a constant weight fraction of ~0.1%. Reprinted from reference [83] with permission from AIP Publishing LLC.

All of the above discussion shows that laser-induced sample heating effects have to be taken into account in any analysis of composite properties by Raman spectroscopy. This is particularly true for the analysis of the response of a composite filled with carbon species submitted to a strain, since some Raman bands of carbon fibers, especially the G′ band, which exhibits a rate of frequency shift twice that of the D band, have been seen to shift to lower frequencies with increasing uniaxial strain [82]. The rate of band shift of the G′ band of epoxy/SWCNT composites was found to be −6.0 and −6.8 cm^−1^/% strain for composites cured under two different conditions, the difference may be due to the strength of the interface of the polymer–filler systems [79]. According to the authors, the downshifts indicate stress transfer between the epoxy matrix and the nanotubes. At high strains (>0.5%), the shift reaches a plateau level, which is supposed to be due to interfacial failure. In a study carried out on PDMS filled with 0.1 wt % of graphene platelets (GPL), Srivastava et al. [83] showed that, below 1.5% strain, the G mode peak of GPL reduces in frequency with applied tensile strain with a rate of peak shift with a strain around 2.4 cm^−1^/composite strain % which is much higher than that (~0.1 cm^−1^/composite strain%) found for a SWCNT/PDMS composite. A shift of ~7.3 cm^−1^/composite strain % is reported for GPL in a higher modulus polystyrene (PS) matrix (Figure 9). Figure 9 also displays data under compressive loading. On the basis of the results in Figure 9, the authors suggest that load transfer at the GPL/PDMS interface appears to be more effective in comparison with the SWCNT/PDMS interface. It is also shown that GPL debonds from the PDMS matrix for strain levels >~7%. The larger peak shift in PS is attributed to its higher modulus. Frogley et al. [84] also report a very low magnitude of the spectral shift of the G′ band (2 cm^−1^ over 50% strain) of a SWCNT/PDMS composite with regard to the strain shift of 10–20 cm^−1^ of the SWCNT/epoxy composite.

**Figure 10:**
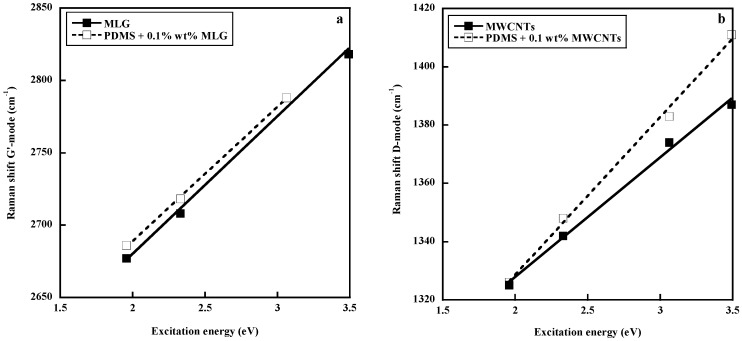
Comparison of the dispersive behavior of multilayer graphene MLG (**a**) and multiwall carbon nanotubes (MWCNTs) (**b**) in the pure state and embedded in a poly(dimethylsiloxane) (PDMS) matrix. Reprinted with permission from Elsevier. Reproduced by permission of Elsevier from L. Bokobza, J.-L. Bruneel, M. Couzi, Vibrational Spectroscopy, 2014, 74, 57–63.

Another approach to get insight into the polymer–filler interface has been to compare the dispersive behavior of the carbon materials in the pure state and embedded in a polymer matrix. In fact, one specific feature in the Raman spectra of carbon species is the shift of the D and G′ bands to higher frequencies with increasing laser excitation energy. The dispersive behavior consists of an upshift around 50 cm^−1^/eV for the D band and around 100 cm^−1^/eV for the G′ band. Since these peculiarities of the Raman spectra of carbon materials arise from their electronic properties, these properties and thus the excitation–energy dependencies of the D and G′ bands are expected to be affected by interfacial interactions between the polymer and the filler. This approach, widely discussed elsewhere [29,76,85,86], contradict the conclusions of Srivastava et al. [83], since the dispersive behavior (slope of the curve representing the wavenumber of the D or G′ bands against the excitation energy) of a multilayer graphene (MLG) in PDMS is quite similar to that of pure MLG (Figure 10a), indicating a poor polymer–filler interface. On the contrary, MWCNTs embedded in PDMS exhibit a higher slope than in the pure state, reflecting an effect of the surrounding polymer on the electronic structure of the filler. It has to be pointed out that PDMS has been shown to exhibit an unexpected affinity for MWCNTs, which seems to be connected to strong CH–π interactions between the PDMS methyl groups and the π–electron-rich surface of the carbon nanotube [87]. Due to the high flexibility of the PDMS chains, a wrapping process around the tube surface is very likely to occur, as predicted by modeling studies [87]. The unprecedented electrical and mechanical properties of PDMS/CNT composites [84,87,88,89] probably originate from the unique adsorption properties of PDMS chains on the CNT surface explained by the CH–π bonding.

Let us note that in parallel to the AFM-IR mentioned above, tip-enhanced Raman spectroscopy (TERS), which combines Raman spectroscopy and scanning probe microscopy in order to achieve nanometer-scale spatial resolution, thus overcomes the diffraction limit of the conventional Raman spectroscopy. In the TERS technique, a sharp metal tip is focused at the center of a laser beam and can be precisely positioned at different regions on the surface of the sample. A strong confinement and enhancement of the electromagnetic field observed at the tip-apex arising from a combination of surface plasmon resonance and antenna effects, enhances the Raman signal from the molecules in the vicinity of the apex of the scanning probe. The reader will find further details and recent advances of this technique in various areas of applications in the literature [90,91]. An interesting illustration can be found in a paper by Saito and Yanagi [92], in which the TERS spectra of β-carotene encapsulated in SWCNTs were performed in 100 nm steps along bundles of single tubes aligned parallel to each other. From the absence of β-carotene in some spectra, it was demonstrated that the rate of encapsulation of β-carotene was not uniform, which could have occurred if the tubes were twisted during encapsulation or were filled with impurities. This points out that the main advantage of TERS is to avoid the averaging of Raman spectra of nanocomposite materials that are not spatially uniform. Furthermore, nanoscale uniaxial pressure of a tip on an isolated SWCNT bundle was investigated by TERS and the shifts in peak position and changes in Raman intensity were related to the tube deformation caused by the tip pressure [93]. In another paper by Yano et al. [94], it was demonstrated that TERS-based nanoimaging technique can reveal a strain distribution along the length of isolated CNTs that were manipulated into various shapes by the AFM-tip dragging method.

The usefulness of TERS in studying the structure and interactions of various kinds of carbon nanotube-based and graphene-based nanomaterials is demonstrated in a paper by Vantasin et al. [95]. In SBR/MWCNT composites, for example, TERS spectra were measured at different locations and marked position-dependent spectral variations were observed with changes in the relative intensities of the MWCNT and SBR bands as well as in the relative intensities of the bands associated with vinyl configurations of the butadiene phase and those related to the styrene units [96]. The observed results lead the authors to suggest that the local distribution of the polymer chains is modified with changes in the orientation of the phenyl rings by π–π interactions between the polymer chains and the carbon nanotubes.

The above examples demonstrate that TERS characterization provides insights into localized variations of structural properties in nanomaterials, which opens the way for the analysis of polymer–filler interfaces at a high spatial resolution far beyond the diffraction limit of light.

It is also useful to mention the use of polymer–metal nanocomposites as surface-enhanced Raman scattering (SERS) substrates [97,98,99,100]. SERS is a molecular spectroscopic technique that enhances the Raman intensity of molecules adsorbed on a rough metal surface typically of silver or gold [101]. This technique is particularly useful for a characterization of various surfaces at a molecular level. In the work of Giesfelt et al. [97], the physical vaporization of gold on PDMS provides a rapid diffusion of the metal particles and yields nanocomposites with unique tunable surface plasmon resonance characteristics. The spectra of *n*-phenyl-1,2-diphenylene diamine on Au-PDMS has been shown to be more intense than that on a traditional Au-glass SERS substrate. On the other hand, the σ or π-orientation of the molecule to the metal particles is suggested to explain the different band enhancements of the two SERS substrates. Biswas et al. [98] have demonstrated that polymer–metal fractal nanocomposites formed just below the percolation threshold are stable substrates that display a large enhancement in the SERS signal. Fateixa et al. [99] carried out SERS measurements of Ag/poly(butyl) acrylate nanocomposites below and above the glass transition temperature of the composite material, because the mobility of the polymer chains is expected to affect the contact between the adsorbate and the metal surface. It was observed that a change in polymer mobility has no marked effect on the SERS signal of the analyte. The approach of Rao et al. [100] is to use polymer films with bimetallic nanoparticles of Ag-Au to optimize the SERS response. By increasing the Au content of the Ag-poly(vinyl alcohol) film, all the peaks of the analyte molecule (Rhodamine 6G) are seen to increase, reach a maximum, then decrease significantly.

Finally, it is of interest to report an interesting application of graphene in the field of surface-enhanced Raman scattering (SERS). Some molecules, especially aromatic, can be adsorbed on the flat surface of the graphene sheet leading to an enhancement of the Raman signal of the adsorbed molecules in the case of strong interactions between the substrate and the molecules, thus promoting a charge transfer [102]. Metal nanoparticles have also been coupled with graphene to develop what is called a graphene-mediated surface-enhanced Raman scattering [103].

## 5. Conclusions

The importance of various spectroscopic techniques for investigating polymer composites were discussed in this review. While fluorescence spectroscopy, contrary to infrared and Raman spectroscopy, cannot provide detailed information on molecular structures, the fluorescence resonance energy transfer between two chromophores is an important phenomenon for the analysis of polymer–filler interfaces. The fluorescence behavior of small molecules can be used to probe confinement effects in filled systems or intercalation and exfoliation in clay nanocomposites. Solid-state NMR can identify the silanol hydroxyl groups present on the silica surface and analyze their interactions with the polymer chains. Moreover, solid-state NMR is probably one of the most effective tool for the analysis, through the determination of the relaxation times, of the dynamics of polymer chains at the interfacial region of filled systems. Over the years, infrared spectroscopy has been extensively used to characterize a wide range of materials through bands associated with the functional groups of the fillers or the polymers. Near-infrared spectroscopy, which probes overtones and combination bands, can be used to analyze the chemical groups that display strong absorptions in the mid-infrared. It is particularly interesting for the determination of chain orientation in the case of thick samples analyzed in transmission. Raman spectroscopy has become a key technique for the characterization of carbon-based materials that display, due to resonance-enhanced scattering effects, strong, well-defined bands even if used at very small amounts in the composite. Combined with scanning probe microscopy, Raman spectroscopy has been launched to a new dimension with unprecedented insights into phenomena relevant to the field of nanomaterials. Nevertheless, it should be recalled that the laser power must be controlled in order to avoid sample heating that can shift the Raman bands.
